# Generally weighted moving average control chart in the presence of measurement error via auxiliary information utilization

**DOI:** 10.1371/journal.pone.0333278

**Published:** 2025-09-30

**Authors:** Jen-Hsiang Chen, Kashinath Chatterjee, Shin-Li Lu, Su-Fen Yang

**Affiliations:** 1 Department of Information Management, Shih Chien University Kaohsiung Campus, Kaohsiung City, Taiwan; 2 Department of Population Health Sciences, Division of Biostatistics and Data Science, Medical College of Georgia, Augusta University, Augusta, Georgia, United States of America; 3 Department of Industrial and Systems Engineering, Chung Yuan Christian University, Taoyuan City, Taiwan; 4 Department of Statistics, National Chengchi University, Muzha, Taipei, Taiwan; Sepuluh Nopember Institute of Technology: Institut Teknologi Sepuluh Nopember, INDONESIA

## Abstract

Control charts are essential tools for monitoring the stability of manufacturing processes. However, measurement error can reduce their effectiveness by weakening their ability to detect process shifts. This study introduces an improved version of the Generally Weighted Moving Average (GWMA) chart, called the Auxiliary Information Based GWMA with Measurement Error (AIB-GWMA-ME) chart. This new chart combines auxiliary information with a measurement error adjustment mechanism to improve monitoring accuracy. Three types of measurement error models are considered – namely, the covariate model, multiple measurements model, and linearly increasing variance model. For each model, the statistic of the AIB-GWMA-ME chart is developed, and the corresponding control limits are determined. Monte Carlo simulations are used to assess the chart’s performance based on Average Run Length (ARL). Results show that the AIB-GWMA-ME chart improves sensitivity to small shifts and performs better than existing GWMA and EWMA charts in the presence of measurement error.

## 1. Introduction

Product quality largely depends on production process stability. Statistical Process Control (SPC) uses real-time statistical methods to detect abnormal variations and support timely corrective actions. Control charts, the most common SPC tools, help monitor process shifts, reduce variability, and maintain consistent quality.

Shewhart introduced control charts in 1924 to detect large process shifts due to assignable causes. While effective initially, their limitations in identifying small shifts became evident with advancements in precision manufacturing. To address this, Roberts [1] developed the Exponentially Weighted Moving Average (EWMA) chart, which improved sensitivity by incorporating both current and past data. In addition to this, Sheu and Lin [2] introduced the Generally Weighted Moving Average (GWMA) chart. Simulation results showed that the GWMA chart outperformed the EWMA chart in terms of shift detection capability. When the design and adjustment parameters were set to q=1−λ and α=1, the EWMA chart could be considered a special case of the GWMA chart.

Control charts are primarily intended to detect process shifts caused by assignable sources. However, shifts introduced by measurement error (ME)-the discrepancy between the true value of a quality characteristic and its observed value-are often overlooked. Using such inaccurate measurements in control chart construction can distort the true process state, compromising monitoring accuracy and leading to incorrect decisions. As a result, the adverse effects of measurement error on control chart performance have received increasing research attention.

Following Mittag and Stemann [3], Linna and Woodall [4] developed a measurement error model and demonstrated its detrimental impact on the efficiency of process monitoring. Maravelakis et al. [5] investigated the impact of measurement error on EWMA charts using a covariate model and improved its performance through a multiple measurement approach. Further developments on similar control charts have been explored by several researchers, including Abbasi [6], Daryabari et al. [7], Cheng and Wang [8], Salmasnia et al. [9], Tang et al. [10], Noor-ul-Amin et al. [11], Asif et al. [12], Wang et al. [13], Sarwar et al. [14], Chen et al. [15], Zaagan et al. [16], and Ahmadini et al. [17].

To improve monitoring efficiency, many researchers have incorporated one or more auxiliary quality characteristics alongside the primary variable to construct more effective control charts. This approach, known as the Auxiliary Information Based (AIB) control chart, aims to enhance parameter estimation accuracy and, in turn, the overall monitoring performance.

Riaz [18] initiated the application of regression estimators as charting statistics for monitoring process variability and demonstrated through simulation results that the proposed chart outperformed the traditional range and variance Shewhart charts. In addition, Riaz [19] introduced the AIB-Shewhart mean chart, which showed superior detection efficiency compared to the traditional Shewhart mean chart. Abbas et al. [20] proposed the AIB-EWMA chart and shown that the proposed chart performs better than the EWMA chart for detecting small shifts. To further enhance monitoring performance, mention may be made to Haq [21], and Javaid et al. [22]. Recently, Haq and Abidin [23] integrated the feature of the GWMA scheme with the AIB-EWMA chart to propose the AIB-GWMA chart, and confirmed that its control chart effectively improves the ability to monitor small process deviations.

Building on this motivation, this study examines the impact of measurement errors on the AIB-GWMA chart, hereafter referred to as the AIB-GWMA-ME chart. This proposed approach not only retains the GWMA chart’s superior sensitivity to small process shifts, but also exploits auxiliary information to improve estimation accuracy and introduces correction mechanisms that effectively counteract the detrimental effects of measurement errors

**Fig 1:**
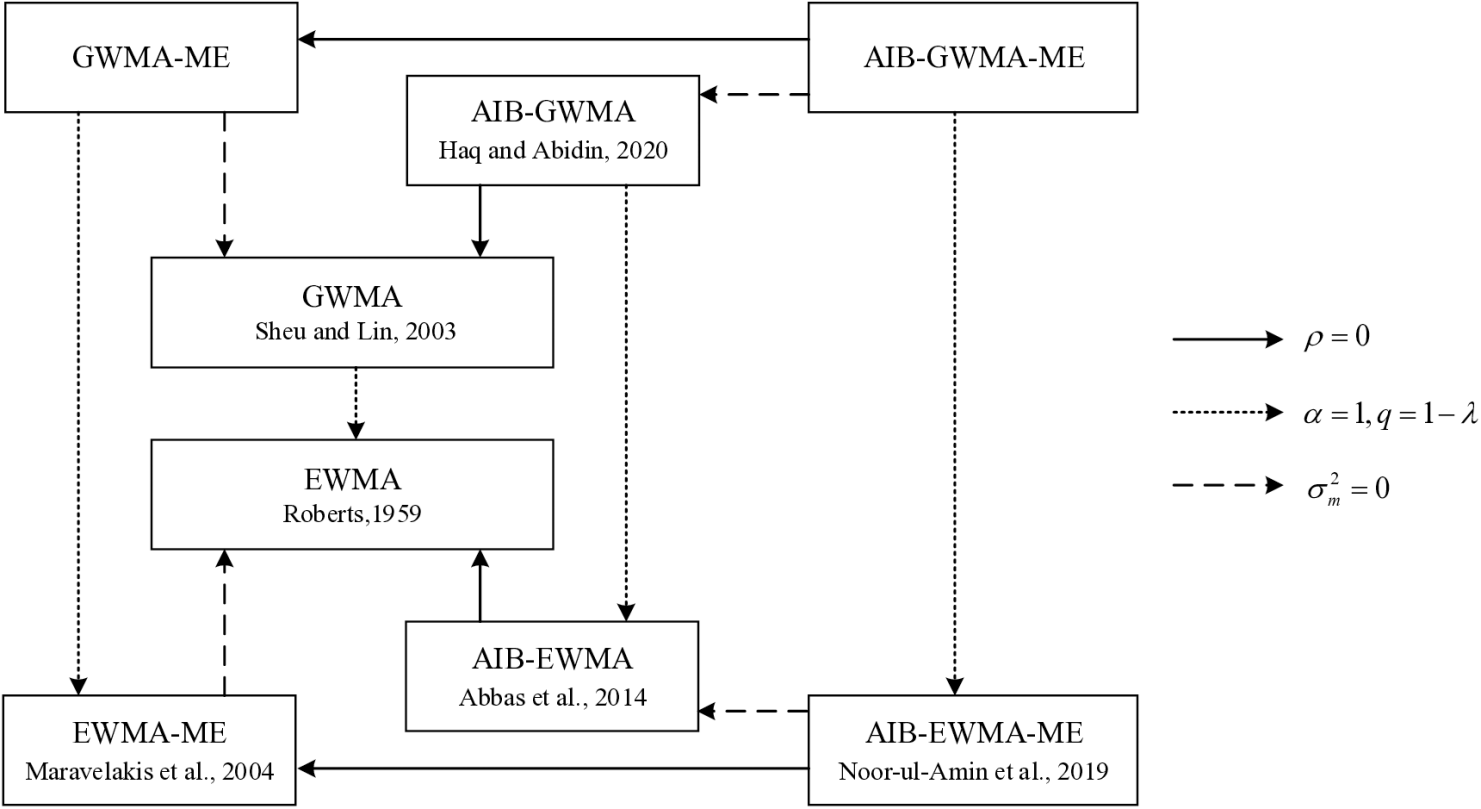
The relationship diagram of various control charts.

To assess the proposed method, this study will develop an R program and perform extensive Monte Carlo simulations. These simulations will estimate the Average Run Length (ARL) under three types of measurement error - covariate error, multiple measurements, and linearly increasing variance - across various levels of process shift. The performance of the proposed control chart will be compared with existing control charts - including EWMA, GWMA, AIB-EWMA, AIB-GWMA, and AIB-EWMA-ME - focusing on detection efficiency and stability to demonstrate its overall effectiveness and robustness. The relevant simplified and relationship diagram is shown in Fig 1.

## 2. The GWMA-ME chart

Since changes caused by measurement errors may have adverse effects on the monitoring capabilities of control charts, it has attracted many scholars to investigate this field. Maravelakis et al. [5] examined the performance of the EWMA chart under three distinct measurement error models: covariate measurement errors, multiple measurements, and linearly increasing variance. The findings indicate that measurement error significantly impacts the monitoring efficiency of the EWMA chart. To enhance its ability to detect small process shifts, the study incorporates the properties of the GWMA chart, which outperforms the EWMA chart in identifying small shifts. Consequently, a GWMA chart that accounts for measurement error, referred to as the GWMA-ME chart, is developed.

### 2.1. GWMA-ME chart using covariate model

Suppose that the process is in control, and the study variable X follows a normal distribution with mean μ and variance $\sigma^{2}$. However, the variable of interest Y is a covariate of the study variable X and their relationship can be expressed using the covariate model:


Y=A+BX+ε, (1)

where A and B are known constants while ε is the random error term which is independent of X and is normally distributed with mean 0 and variance $\sigma_{m}^{2}$. IfA=0 and B=1, the covariate model will reduce to the usual additive error model by Bennet [24]. Since the quality characteristic X is normally distributed, its covariate Y also follows a normal distribution with mean A+Bμ and variance $B^{2}\sigma^{2}+\sigma_{m}^{2}$. That is, $Y\sim N(A+B\mu ,B^{2}\sigma^{2}+\sigma_{m}^{2})$.

Sheu and Lin [2] introduced an adjustment parameter to enhance the EWMA chart, extending it into the GWMA scheme, which significantly improved its detection performance. Assume $Y_{it}$, i=1,2,…,n, are independent quantities of size *n* measured at time t, t=1,2,.... Let ${\bar{Y}}_{t}$ be the sample mean of the t−th subgroup, where ${\bar{Y}}_{t}=\sum {\mathrm{\backslash nolimits}}_{i=1}^{n}Y_{it}/n$ are independent normal random variables with mean A+Bμ and variance $(B^{2}\sigma^{2}+\sigma_{m}^{2})/n$. According to Sheu and Lin [2], Let $G_{t}^{1}$ denotes the GWMA-ME, using covariate model, charting statistics at time t. Then, $G_{t}^{1}$ can be expressed as:


$$G_{t}^{1}=\sum_{j=1}^{t}(q^{{\left(j-1\right)}^{\alpha }}-q^{j^{\alpha }}){\bar{Y}}_{t-j+1}+q^{j^{\alpha }}G_{0}^{1}$$ (2)

where the design parameter q is a constant satisfying 0≤q<1, and the adjustment parameter α is also a constant satisfying 0<α≤1. Moreover, α is used to determine the kurtosis of the weighting function. The starting value $G_{0}^{1}$ is usually set as the mean of ${\bar{Y}}_{t}$, that is, $G_{0}^{1}=A+B\mu$.

The expected value of $G_{t}^{1}$ can be calculated as


$$E(G_{t}^{1})=E\left(\sum_{j=1}^{t}(q^{{\left(j-1\right)}^{\alpha }}-q^{j^{\alpha }}){\bar{Y}}_{t-j+1}+q^{j^{\alpha }}G_{0}^{1}\right)=A+B\mu$$ (3)

The variance of $G_{t}^{1}$ is


$$Var(G_{t}^{1})=Var\left(\sum_{j=1}^{t}(q^{{\left(j-1\right)}^{\alpha }}-q^{j^{\alpha }}){\bar{Y}}_{t-j+1}+q^{j^{\alpha }}G_{0}^{1}\right)=Q_{t}\cdot \frac{B^{2}\sigma^{2}+\sigma_{m}^{2}}{n}$$ (4)

where $Q_{t}=\underset{t\to \infty }{\lim}\left(\sum_{j=1}^{t}{\left(q^{{\left(j-1\right)}^{\alpha }}-q^{j^{\alpha }}\right)}^{2}\right)$. Define $Q_{\infty }=\underset{t\to \infty }{\lim}\;Q_{t}$, which is the asymptotic variance of the GWMA-ME statistic. Now, if $L^{1}$ denotes the width of the control limit, the GWMA-ME chart using a covariate model with asymptotic control limits can be represented as


$$\left\{ \;\;\begin{array}{c} UCL=A+B\mu +L^{1}\sqrt{\left((B^{2}\sigma^{2}+\sigma_{m}^{2})/n\right)\cdot Q_{\infty }}\\ CL=A+B\mu \\ LCL=A+B\mu -L^{1}\sqrt{\left((B^{2}\sigma^{2}+\sigma_{m}^{2})/n\right)\cdot Q_{\infty }} \end{array}\right.$$ (5)

If the GWMA-ME charting statistic $G_{t}^{1}$ lies between the control limits LCL and UCL, the process will be in control. However, if $G_{t}^{1}>UCL$ or $G_{t}^{1}<LCL$, the process will be said to be out of control, which means there is a shift in the process parameter. In particular, for α=1 and q=1−λ, Eq. (2) reduces to the well-known EWMA statistic presented in Maravelakis et al. [5], and can be transformed into


$$E_{t}=\lambda \sum_{j=1}^{t}{\left(1-\lambda \right)}^{j-1}{\bar{Y}}_{t-j+1}+{\left(1-\lambda \right)}^{t}E_{0},\;E_{0}=A+B\mu$$ (6)

The mean and variance of the EWMA statistic $E_{t}$ are A+Bμ and $\lambda (B\sigma^{2}+\sigma_{m}^{2})/n(2-\lambda )$. That is, the EWMA chart is a special case of the GWMA-ME chart using a covariate model when α=1.

### 2.2. GWMA-ME chart using multiple measurements

To minimize the impact of measurement errors on the variable of interest, Linna and Woodall [4] recommended performing multiple measurements of quality characteristics at each sampling time. However, adopting this technique requires careful consideration of the associated sampling costs and time. Maravelakis et al. [5] adopted the average of multiple measurements to explore the effect of it on the EWMA chart. According to the covariate model in Eq. (1), this paper extends the EWMA chart using multiple measurements to the GWMA-ME chart, and the charting statistic is given by


$$G_{t}^{2}=\sum_{j=1}^{t}(q^{{\left(j-1\right)}^{\alpha }}-q^{j^{\alpha }}){\bar{\bar{Y}}}_{t-j+1}+q^{j^{\alpha }}G_{0}^{2}$$ (7)

where the design parameter q and adjustment parameter α are the same as described earlier, ${\bar{\bar{Y}}}_{t}$ is the mean of the multiple measurements collected at a time t. The starting value $G_{0}^{2}$ is as the mean of ${\bar{\bar{Y}}}_{t}$, that is, $G_{0}^{2}=A+B\mu$. If $L^{2}$ denotes the width of the control limit, the GWMA-ME chart using multiple measurements with asymptotic control limits can be represented as


$$\left\{ \;\;\begin{array}{c} UCL=A+B\mu +L^{2}\sqrt{\left(B^{2}\sigma^{2}/n+\sigma_{m}^{2}/nk\right)\cdot Q_{\infty }}\\ CL=A+B\mu \\ LCL=A+B\mu -L^{2}\sqrt{\left(B^{2}\sigma^{2}/n+\sigma_{m}^{2}/nk\right)\cdot Q_{\infty }} \end{array}\right.$$ (8)

where k measurements are collected from each selected unit or sample. When the GWMA-ME statistic $G_{t}^{2}$ remains within the control limits between LCL and UCL, the process is considered to be in control. However, if the statistic falls outside LCL or UCL it indicates that the process has shifted.

### 2.3. GWMA-ME chart using linearly increasing variance

In the covariate model, it is assumed that the variance of the measurement error remains constant. However, this assumption is often unrealistic in industrial settings. Linna and Woodall [4], as well as Maravelakis et al. [5], proposed a technique involving linearly increasing variance to account for situations where the variance of the covariate model changes linearly with the mean level of the process. This means the measurement error ε is assumed to follow a normal distribution with mean 0 and variance C+Dμ, whereas C and D are known constants. Accordingly, the variable of interest Y follows a normal distribution with mean A+Bμ and variance $B^{2}\sigma^{2}+C+D\mu$. That is, $Y\sim N(A+B\mu ,B^{2}\sigma^{2}+C+D\mu )$.

Based on the statistic in Eq. (2), the GWMA-ME chart using linearly increasing variance with asymptotic control limits can be represented as


$$\left\{ \;\;\begin{array}{c} UCL=A+B\mu +L^{3}\sqrt{\left((B^{2}\sigma^{2}+C+D\mu )/n\right)\cdot Q_{\infty }}\\ CL=A+B\mu \\ LCL=A+B\mu -L^{3}\sqrt{\left((B^{2}\sigma^{2}+C+D\mu )/n\right)\cdot Q_{\infty }} \end{array}\right.$$ (9)

Where $L^{3}$ is the width of the control limit.

If the GWMA-ME statistic $G_{t}^{1}$ remains within the control limits between LCL and UCL, the process will be considered to be in control, while if $G_{t}^{1}>UCL$ or$G_{t}^{1}<LCL$, the process will be considered to be out of control.

## 3. The proposed AIB-GWMA-ME chart

Recent research highlights the use of auxiliary information (variables) to improve the detection performance of control charts. Abbas et al. [20] and Haq and Abidin [23] respectively introduced the AIB-EWMA chart and the AIB-GWMA chart, demonstrating that these charts outperform the classical EWMA chart in detecting small process shifts. Moreover, Noor-ul-Amin et al. [11] analyzed the impact of three measurement error models on the AIB-EWMA chart and demonstrated that, with appropriately chosen smoothing constant, the adverse effects of measurement errors on its detection performance can be effectively mitigated. Extending prior research, this study introduces the AIB-GWMA-ME chart and conducts a comprehensive investigation of the impact of measurement errors on the AIB-GWMA framework.

Suppose that there exists an auxiliary variable Z that is correlated with the variable Y of interest, and the correlation coefficient of these two variables is denoted by ρ. Generally, assuming that the expected value and variance of the auxiliary variable are known as $\mu_{Z}$ and $\sigma_{Z}^{2}$, respectively. The paired observations Y and Z are collected in each sample and follow a bivariate normally distributed process $\left(Y,ZsimN_{2}(\mu_{Y},\mu_{Z},\sigma_{Y}^{2},\sigma_{Z}^{2},\rho \right)$. Suppose $\left(Y_{tj},Z_{tj}\right)$, j=1,2,…,n, is a random sample of size n taken from the process at time t, for t=1,2,.... The sample means and variances based on $\left(Y_{t1},Y_{t2},...,Y_{tn}\right)$ and $\left(Z_{t1},Z_{t2},...,Z_{tn}\right)$, respectively, are:


$${\bar{Y}}_{t}=\sum {\mathrm{\backslash nolimits}}_{j=1}^{n}Y_{tj}/n,\;and\;{\bar{Z}}_{t}=\sum {\mathrm{\backslash nolimits}}_{j=1}^{n}Z_{tj}/n$$

$$S_{Y,t}^{2}=\sum {\mathrm{\backslash nolimits}}_{j=1}^{n}(Y_{tj}-{\bar{Y}}_{t}{)}^{2}/(n-1),\;and\;S_{Z,t}^{2}=\sum {\mathrm{\backslash nolimits}}_{j=1}^{n}(Z_{tj}-{\bar{Z}}_{t}{)}^{2}/(n-1)$$

Assuming the underlying process is in control, and following Haq and Khoo [25], the regression estimator of $\mu_{Y}$ is given as:


$$D_{Y,t}^{1}={\bar{Y}}_{t}+\rho \left(\frac{\sigma_{Y}}{\sigma_{Z}}\right)(\mu_{Z}-{\bar{Z}}_{t})$$ (10)

With its mean and variance given by


$$E(D_{Y,t}^{1})=\mu_{Y}\;and\;Var(D_{Y,t}^{1})=\sigma_{D}^{2}=\frac{\text{1}}{n}\sigma_{y}^{2}(1-\rho^{2})\;$$ (11)

### 3.1. AIB-GWMA-ME chart using covariate model

According to the covariate technique we discussed in Section 2.1, the variable of interest Y is normally distributed with mean A+Bμ and variance $B^{2}\sigma^{2}+\sigma_{m}^{2}$. Based on the statistic in Eq. (10), the AIB-GWMA-ME chart using a covariate model with asymptotic control limits can be represented as


$$\left\{ \;\;\begin{array}{c} UCL=A+B\mu +L^{1*}\sqrt{\left(\frac{\text{1}}{n}\left(B^{2}\sigma^{2}+\sigma_{m}^{2}\right)\left(1-\rho^{2}\right)\right)\cdot Q_{\infty }}\\ CL=A+B\mu \\ LCL=A+B\mu -L^{1*}\sqrt{\left(\frac{\text{1}}{n}\left(B^{2}\sigma^{2}+\sigma_{m}^{2}\right)\left(1-\rho^{2}\right)\right)\cdot Q_{\infty }} \end{array}\right.$$ (12)

where $L^{1*}$ is the width of the control chart and $Q_{\infty }=\underset{t\to \infty }{\lim}\left(\sum_{j=1}^{t}{\left(q^{{\left(j-1\right)}^{\alpha }}-q^{j^{\alpha }}\right)}^{2}\right)$, which is the asymptotic variance of the AIB-GWMA-ME statistic.

When the regression estimator $D_{Y,t}^{1}$ remains within the control limits between LCL and UCL, the process will be in control, while if $D_{Y,t}^{1}>UCL$ or $D_{Y,t}^{1}<LCL$, the process will be declared as out of control. Note that when α=1 and q=1−λ, the AIB-GWMA-ME chart will reduce to the case of the AIB-EWMA chart using the covariate model as indicated in Noor-ul-Amin et al. [11]. However, there are typos in the control limits Eqs. (19) and (20) in Noor-ul-Amin et al. [11]. The correct control limits of the AIB-EWMA-ME charts using the covariate model should be as follows:


$$\left\{ \;\;\begin{array}{c} UCL=A+B\mu +L^{1*}\sqrt{\frac{\lambda }{2-\lambda }\cdot \left(\frac{\text{1}}{n}\left(B^{2}\sigma^{2}+\sigma_{m}^{2}\right)\left(1-\rho^{2}\right)\right)}\\ CL=A+B\mu \\ LCL=A+B\mu -L^{1*}\sqrt{\frac{\lambda }{2-\lambda }\cdot \left(\frac{\text{1}}{n}\left(B^{2}\sigma^{2}+\sigma_{m}^{2}\right)\left(1-\rho^{2}\right)\right)} \end{array}\right.$$ (13)

### 3.2. AIB-GWMA-ME chart using multiple measurements

Based on the multiple measurements method as discussed in Section 2.2, the variable of interest Y is normally distributed with mean A+Bμ and variance $B^{2}\sigma^{2}/n+\sigma_{m}^{2}/nk$. Suppose there is an auxiliary variable Z which is correlated with the variable Y of interest and the correlation coefficient of these two variables is denoted by ρ. Assume that the paired observations Y and Z follow a bivariate normal distribution and regression estimator for $\mu_{Y}$ is given in Eq. (10). Let $L^{\text{2}*}$ be the width of the control limit. The AIB-GWMA-ME chart using multiple measurements method with asymptotic control limits can be represented as


$$\left\{ \;\;\begin{array}{c} UCL=A+B\mu +L^{2*}\sqrt{\left(\frac{\text{1}}{n}\left(B^{2}\sigma^{2}+\frac{\sigma_{m}^{2}}{k}\right)(1-\rho^{2})\right)\cdot Q_{\infty }}\\ CL=A+B\mu \\ LCL=A+B\mu -L^{2*}\sqrt{\left(\frac{\text{1}}{n}\left(B^{2}\sigma^{2}+\frac{\sigma_{m}^{2}}{k}\right)(1-\rho^{2})\right)\cdot Q_{\infty }} \end{array}\right.$$ (14)

where k measurements are collected from each selected unit or sample.

When the regression estimator $D_{Y,t}^{1}$ remains within the control limits between LCL and UCL, the process is in control while, if $D_{Y,t}^{1}>UCL$ or $D_{Y,t}^{1}<LCL$, the process will be declared to be out of control. Similarly, for α=1 and q=1−λ, the AIB-GWMA-ME chart will reduce to the case of the AIB-EWMA chart using the multiple measurements method in Noor-ul-Amin et al. [11]. As mentioned earlier, the correct control limits of the AIB-EWMA-ME charts using the multiple measurements method should be as follows:


$$\left\{ \;\;\begin{array}{c} UCL=A+B\mu +L^{2*}\sqrt{\frac{\lambda }{2-\lambda }\cdot \left(\frac{\text{1}}{n}\left(B^{2}\sigma^{2}+\frac{\sigma_{m}^{2}}{k}\right)(1-\rho^{2})\right)}\\ CL=A+B\mu \\ LCL=A+B\mu -L^{2*}\sqrt{\frac{\lambda }{2-\lambda }\cdot \left(\frac{\text{1}}{n}\left(B^{2}\sigma^{2}+\frac{\sigma_{m}^{2}}{k}\right)(1-\rho^{2})\right)} \end{array}\right.$$ (15)

### 3.3. AIB-GWMA-ME chart using linearly increasing variance

As outlined in Section 2.3, the linearly increasing variance method has been adopted, indicating that the variable of interest Y follows a normal distribution with a mean A+Bμ and variance $B^{2}\sigma^{2}+C+D\mu$. Suppose we have an auxiliary variable Z which is correlated with Y and the correlation coefficient between Y and Z is denoted by ρ. Based on the regression estimator for Y as shown in Eq. (10), and supposing $L^{3*}$ be the width of the control limit is, the AIB-GWMA-ME chart using the linearly increasing variance method with asymptotic control limits can be represented as


$$\left\{ \;\;\begin{array}{c} UCL=A+B\mu +L^{3*}\sqrt{\left(\frac{\text{1}}{n}(B^{2}\sigma^{2}+C+D\mu )(1-\rho^{2})\right)\cdot Q_{\infty }}\\ CL=A+B\mu \\ LCL=A+B\mu -L^{3*}\sqrt{\left(\frac{\text{1}}{n}(B^{2}\sigma^{2}+C+D\mu )(1-\rho^{2})\right)\cdot Q_{\infty }} \end{array}\right.$$ (16)

When the regression estimator $D_{Y,t}^{1}$ remains within the control limits betweenLCL and UCL, the process will be declared as in control, while if $D_{Y,t}^{1}>UCL$ or $D_{Y,t}^{1}<LCL$, the process will be declared as out of control. Similarly, for α=1 and q=1−λ, the AIB-GWMA-ME chart will reduce to the case of the AIB-EWMA chart using the linearly increasing variance method in Noor-ul-Amin et al. [11]. As mentioned earlier, the correct control limits of the AIB-EWMA-ME charts using the linearly increasing variance method should be as follows:


$$\left\{ \;\;\begin{array}{c} UCL=A+B\mu +L^{3*}\sqrt{\frac{\lambda }{2-\lambda }\cdot \left(\frac{\text{1}}{n}(B^{2}\sigma^{2}+C+D\mu )(1-\rho^{2})\right)}\\ CL=A+B\mu \\ LCL=A+B\mu -L^{3*}\sqrt{\frac{\lambda }{2-\lambda }\cdot \left(\frac{\text{1}}{n}(B^{2}\sigma^{2}+C+D\mu )(1-\rho^{2})\right)} \end{array}\right.$$ (17)

The symbols and acronyms used in this study are summarized as follows. The detailed list is presented in Table A1 in the S1 Appendix.

## 4. Performance of the AIB-GWMA-ME chart

This section evaluates the performance of the proposed AIB-GWMA-ME chart under various measurement error scenarios and compares its detection capability with that of existing control charts. The primary performance metric used is the Average Run Length (ARL), which indicates a chart’s sensitivity to small shifts in the process mean. For a comprehensive assessment, the analysis is organized around three measurement error frameworks: the covariate model, the multiple measurements method, and the linearly increasing variance method.

Each specific combination of control parameters is followed by simulated ARL results. Comparative analyses are conducted between AIB-GWMA-ME and other relevant control charts, such as GWMA-ME, EWMA-ME, AIB-EWMA-ME, and AIB-EWMA, focusing on detection performance, robustness to measurement error, and parameter stability. Through these analyses, the effectiveness and adaptability of the proposed AIB-GWMA-ME chart are verified across various error conditions, providing critical support for its practical applicability.

Table 1 presents the ARL values of the AIB-GWMA-ME chart under the covariate model with constants and parameters set as A=0,B=1 and q=0.95, against various correlation coefficients ρ, adjustment parameters α, and measurement error ratios $\sigma_{m}^{2}/\sigma^{2}$. When the process remained in-control (δ=0), all combinations of parameter settings yielded ARL values close to 370, indicating strong stability and a low false alarm rate for all control charts. However, when process shifts occurred (δ>0), ARL values decreased accordingly, confirming the sensitivity of the control charts to shifts and enabling further analysis of their performance under various conditions. Table 1 indicates that the AIB-GWMA-ME chart demonstrated superior monitoring performance against a wide range of shift magnitudes and existing measurement errors.

**Table 1:** ARL values for the AIB-GWMA-ME charts with covariate model by taking A=0, B=1, q=0.95 when $ARL_{0}\approx 370$.

	ρ=0				ρ=0.25				ρ=0.5				ρ=0.75				ρ=0.95			
α	0.5	0.7	0.9	1.0	0.5	0.7	0.9	1.0	0.5	0.7	0.9	1.0	0.5	0.7	0.9	1.0	0.5	0.7	0.9	1.0
$\sigma_{m}^{2}/\sigma^{2}=0$																				
$\delta \backslash L^{1*}$	2.766	2.572	2.495	2.489	2.767	2.569	2.495	2.490	2.765	2.568	2.496	2.488	2.764	2.569	2.495	2.488	2.769	2.567	2.496	2.491
0.00	370.24	370.31	370.04	370.33	370.05	369.79	369.87	369.87	370.58	369.72	369.82	369.56	370.81	369.97	370.65	369.84	370.09	369.69	369.99	370.17
0.10	88.19	**80.26**	**81.78**	85.95	84.68	**77.02**	**78.22**	82.34	73.76	**67.25**	**67.57**	70.11	52.02	47.30	**46.13**	46.89	18.55	17.46	16.66	16.26
0.25	26.32	24.34	23.06	22.65	**25.18**	23.31	22.11	21.70	21.48	20.12	19.12	18.63	14.59	14.09	13.57	13.21	4.81	5.27	5.57	5.59
0.50	9.68	9.75	9.59	9.40	9.23	9.31	9.23	9.06	7.82	8.05	8.09	7.95	5.21	5.68	5.92	5.93	1.74	2.27	2.69	2.85
1.00	3.44	3.99	4.35	4.44	3.29	3.83	4.20	4.30	2.79	3.33	3.74	3.85	1.88	2.42	2.84	3.00	1.00	1.02	1.41	1.79
$\sigma_{m}^{2}/\sigma^{2}=0.1$																				
$\delta \backslash L^{1*}$	2.766	2.572	2.495	2.489	2.767	2.569	2.495	2.49	2.764	2.568	2.497	2.489	2.764	2.569	2.494	2.489	2.769	2.569	2.496	2.492
0.00	370.24	370.31	370.04	370.33	370.05	369.96	369.84	369.92	369.88	370.15	370.66	370.12	370.11	369.78	369.92	369.97	369.73	370.18	370.15	370.25
0.10	88.64	**80.90**	**82.27**	86.63	85.20	**77.45**	**78.69**	82.86	74.14	**67.63**	**68.10**	70.67	52.40	47.62	**46.40**	47.22	18.70	17.59	16.77	16.36
0.25	26.50	24.51	23.20	22.79	25.37	23.46	22.24	21.85	21.61	20.25	19.24	18.76	14.69	14.18	13.65	13.30	4.84	5.31	5.59	5.62
0.50	9.76	9.82	9.66	9.45	9.30	9.38	9.29	9.11	7.87	8.10	8.14	8.00	5.25	5.72	5.96	5.96	1.75	2.28	2.70	2.87
1.00	3.47	4.02	4.37	4.46	3.31	3.85	4.22	4.32	2.81	3.35	3.76	3.87	1.90	2.44	2.85	3.01	1.00	1.02	1.43	1.80
$\sigma_{m}^{2}/\sigma^{2}=0.5$																				
$\delta \backslash L^{1*}$	2.766	2.572	2.495	2.489	2.767	2.570	2.498	2.491	2.769	2.574	2.500	2.494	2.783	2.586	2.513	2.508	2.905	2.697	2.620	2.616
0.00	370.24	370.31	370.04	370.33	370.25	369.48	370.50	369.68	369.93	370.47	369.72	370.50	369.78	369.73	370.41	370.20	370.77	370.04	369.76	370.05
0.10	**100.54**	**92.89**	**95.57**	100.63	97.04	**88.52**	**91.59**	96.34	84.77	**77.34**	**78.52**	82.73	60.61	**55.08**	**54.36**	55.87	23.26	21.67	20.55	20.13
0.25	30.60	28.28	26.75	26.38	29.34	27.05	25.70	25.29	25.17	23.36	22.16	21.71	17.27	16.46	15.72	15.32	6.10	6.49	6.66	6.63
0.50	11.45	11.31	11.00	10.74	10.92	10.82	10.59	10.34	9.23	9.36	9.25	9.08	6.23	6.62	6.78	6.74	2.19	2.73	3.15	3.29
1.00	4.06	4.59	4.91	4.97	3.87	4.40	4.75	4.81	3.29	3.84	4.21	4.30	2.24	2.78	3.20	3.34	1.01	1.16	1.80	1.97
$\sigma_{m}^{2}/\sigma^{2}=\text{1}\mathrm{.0}$																				
$\delta \backslash L^{1*}$	2.766	2.572	2.495	2.489	2.774	2.580	2.503	2.496	2.805	2.606	2.531	2.525	2.914	2.707	2.630	2.622	3.704	3.445	3.342	3.335
0.00	370.24	370.31	370.04	370.33	369.70	370.90	369.74	369.83	370.06	369.92	369.78	369.51	370.35	370.08	369.72	369.55	370.26	369.94	369.99	370.36
0.10	**130.52**	**123.28**	**129.56**	137.59	**125.95**	**119.02**	**124.71**	131.87	**112.78**	**105.22**	**109.03**	115.92	86.61	**78.70**	**80.31**	84.51	44.77	40.62	**39.24**	39.62
0.25	42.22	38.37	**36.98**	37.11	**40.61**	36.99	**35.50**	35.59	35.43	32.41	30.84	30.72	25.77	23.83	22.65	22.17	12.43	12.19	11.82	11.52
0.50	16.07	15.44	14.75	14.35	15.41	14.86	14.23	13.84	13.32	12.99	12.57	12.25	9.48	9.55	9.43	9.24	4.42	4.92	5.23	5.27
1.00	5.77	6.20	6.39	6.37	5.53	5.97	6.18	6.17	4.75	5.24	5.52	5.54	3.37	3.91	4.29	4.37	1.60	2.13	2.54	2.71

For a small shift (δ=0.10), the AIB-GWMA-ME chart exhibits the best detection performance. For instance, considering ρ=0.5, α=0.9 and no measurement error $(\sigma_{m}^{2}/\sigma^{2}=0)$, the AIB-GWMA chart has an ARL value of 67.57, which is lower than that of the AIB-EWMA chart (ARL=70.11), indicating that adding the design parameter q effectively enhanced detection sensitivity. When the measurement error increased to $(\sigma_{m}^{2}/\sigma^{2}=1.0)$, the ARL increased to 109.03, reflecting a degradation in detection ability; however, the proposed control chart performed better than the AIB-EWMA-ME chart, indicating relatively greater robustness.

The impact of the correlation coefficient ρ on AIB-GWMA-ME performance also yielded important insights. When ρ=0, the AIB-GWMA-ME chart simplifies to the existing GWMA-ME, relying solely on the primary variable Y. As ρ increased, the ARL values declined significantly, confirming that the inclusion of an auxiliary variable markedly improved detection sensitivity. At a low correlation coefficient ρ=0.25, the AIB-GWMA-ME chart needs less time to detect small process mean shifts than the AIB-EWMA-ME chart. However, at a high correlation coefficient ρ=0.95, the AIB-EWMA-ME chart performs a little better than the AIB-GWMA-ME chart in detecting small shifts. Furthermore for ρ=0.95, the AIB-GWMA-ME consistently achieved the lower ARL values against all shift scenarios, demonstrating a strong correlation between Y and the auxiliary variable Z enhanced monitoring effectiveness.

Also, the AIB-GWMA-ME chart was reduced to the AIB-GWMA chart when the absence of measurement error generally outperformed the AIB-EWMA chart against most conditions. For example, when ρ=0.75,α=0.9, the AIB-GWMA chart had an ARL value of 46.13, which was slightly better than the AIB-EWMA chart with an ARL value of 46.89, indicating stronger detection sensitivity under the GWMA framework when combined with auxiliary variables. Similarly, under measurement error conditions, the AIB-GWMA-ME chart outperformed its AIB-EWMA-ME counterpart. When ρ=0.75,α=0.7, and $\sigma_{m}^{2}/\sigma^{2}=1.0$, the ARL value of the AIB-GWMA-ME chart was 78.70, compared to 84.51 for the AIB-EWMA-ME chart, demonstrating that the GWMA structure maintained better stability and detection performance under measurement error.

To investigate the effects of the constant B, Table 2 shows the ARL values of the AIB-GWMA-ME chart with the covariate model by the same parameters in Table 1 but with various values of B at $\sigma_{m}^{2}/\sigma^{2}=1$. Table 2 shows that the value of B increases, and the ARL values decrease. It can be shown that under ρ=0.25 and α=0.9, the ARL values of the AIB-GWMA-ME chart decrease from 124.71 (B=1) to 82.87 (B=5), and consistently outperforms the corresponding AIB-EWMA-ME chart (α=1). This indicates that increasing B effectively mitigated the negative effects of measurement error and enhanced the detection ability of AIB-based control charts.

**Table 2:** ARL values for the AIB-GWMA-ME charts with covariate model by taking A=0, $\sigma_{m}^{2}/\sigma^{2}=1$, q=0.95 when $ARL_{0}\approx 370$.

	ρ=0				ρ=0.25				ρ=0.5				ρ=0.75				ρ=0.95			
α	0.5	0.7	0.9	1.0	0.5	0.7	0.9	1.0	0.5	0.7	0.9	1.0	0.5	0.7	0.9	1.0	0.5	0.7	0.9	1.0
B=1																				
$\delta \backslash L^{1*}$	2.766	2.572	2.495	2.489	2.774	2.58	2.503	2.496	2.805	2.606	2.531	2.525	2.914	2.707	2.63	2.622	3.704	3.445	3.342	3.335
0.00	370.24	370.31	370.04	370.33	369.70	370.90	369.74	369.83	370.06	369.92	369.78	369.51	370.35	370.08	369.72	369.55	370.26	369.94	369.99	370.36
0.10	**130.52**	**123.28**	**129.56**	137.59	**125.95**	**119.02**	**124.71**	131.87	**112.78**	**105.22**	**109.03**	115.92	86.61	**78.70**	**80.31**	84.51	44.77	40.62	**39.24**	39.62
0.25	42.22	38.37	**36.98**	37.11	40.61	36.99	**35.50**	35.59	35.43	32.41	30.84	30.72	25.77	23.83	22.65	22.17	12.43	12.19	11.82	11.52
0.50	16.07	15.44	14.75	14.35	15.41	14.86	14.23	13.84	13.32	12.99	12.57	12.25	9.48	9.55	9.43	9.24	4.42	4.92	5.23	5.27
1.00	5.77	6.20	6.39	6.37	5.53	5.97	6.18	6.17	4.75	5.24	5.52	5.54	3.37	3.91	4.29	4.37	1.60	2.13	2.54	2.71
B=3																				
$\delta \backslash L^{1*}$	2.766	2.572	2.495	2.489	2.808	2.612	2.534	2.528	2.973	2.765	2.682	2.677	3.5	3.254	3.156	3.149	6.38	5.929	5.753	5.741
0.00	370.24	370.31	370.04	370.33	370.13	370.09	369.94	370.54	370.08	369.87	369.96	370.16	370.42	370.24	369.85	370.16	370.27	369.87	370.16	370.01
0.10	93.90	**85.94**	**88.21**	92.73	91.70	**84.04**	**86.19**	90.50	86.11	**78.41**	**79.58**	83.97	75.56	**68.30**	**68.52**	71.39	62.08	**56.19**	**55.59**	56.93
0.25	28.27	26.10	24.72	24.30	27.64	25.53	24.15	23.72	25.64	23.74	22.51	22.08	22.09	20.69	19.58	19.13	17.85	16.91	16.12	15.72
0.50	10.50	10.45	10.23	10.00	10.21	10.22	10.02	9.80	9.41	9.50	9.37	9.20	8.05	8.27	8.25	8.13	6.41	6.78	6.93	6.87
1.00	3.72	4.26	4.60	4.68	3.63	4.17	4.52	4.60	3.35	3.90	4.26	4.36	2.87	3.42	3.81	3.93	2.30	2.84	3.27	3.40
B=5																				
$\delta \backslash L^{1*}$	2.766	2.572	2.495	2.489	2.824	2.627	2.55	2.543	3.048	2.836	2.751	2.744	3.741	3.478	3.374	3.367	7.302	6.788	6.588	6.57
0.00	370.24	370.31	370.04	370.33	369.79	370.34	370.77	370.71	369.63	370.46	369.85	369.61	369.80	369.74	369.67	369.95	370.05	369.79	369.74	370.22
0.10	90.12	**82.35**	**84.02**	88.43	88.84	**81.17**	**82.87**	87.20	85.31	**77.73**	**78.78**	82.89	78.69	**71.37**	**71.88**	75.10	70.70	**64.18**	**64.10**	66.37
0.25	27.04	24.97	23.66	23.22	26.61	24.61	23.33	22.91	25.34	23.51	22.28	21.85	23.15	21.61	20.49	20.01	20.64	19.37	18.38	17.93
0.50	9.98	10.01	9.82	9.61	9.80	9.86	9.70	9.50	9.28	9.41	9.29	9.11	8.46	8.65	8.58	8.45	7.47	7.76	7.81	7.70
1.00	3.54	4.09	4.44	4.52	3.48	4.03	4.39	4.47	3.31	3.86	4.23	4.32	3.02	3.56	3.95	4.06	2.67	3.22	3.63	3.75

Additionally, as the correlation ρ between the study and auxiliary variables increases from 0.25 to 0.90, the ARL values further decline, enhancing the efficiency of the proposed control chart despite the presence of measurement error. As ρ increased to 0.95, the ARL of the AIB-GWMA-ME chart dropped to 39.24, compared to 39.62 for the AIB-EWMA-ME chart, demonstrating that a high correlation between study and auxiliary variables significantly improved detection sensitivity.

A direct comparison between the AIB-GWMA-ME and AIB-EWMA-ME charts further supported the effectiveness of the former. For example, under B=1,ρ=0.25, and α=0.7, the ARL of the AIB-GWMA-ME chart was 119.02, lower than 131.87 of the AIB-EWMA-ME chart. Similarly, under B=5,ρ=0.5, and α=0.7, the AIB-GWMA-ME chart yields an ARL of 77.73, outperforming the AIB-EWMA-ME chart with an ARL of 82.89. These consistent results highlighted the greater robustness and detection sensitivity of the GWMA framework when combined with AIB and measurement error.

Table 3 presents the ARL values of the AIB-GWMA-ME chart under the multiple measurements model, with constants set as A=0, B=1, k=5 and q=0.95, corresponding various parameter combinations such as correlation coefficient ρ, adjustment parameter α, and measurement error ratio $\sigma_{m}^{2}/\sigma^{\text{2}}$.

**Table 3:** ARL values for the AIB-GWMA-ME charts with multiple measurements method by taking k=5, B=1, q=0.95 when $ARL_{0}\approx 370$.

	ρ=0				ρ=0.25				ρ=0.5				ρ=0.75				ρ=0.95			
α	0.5	0.7	0.9	1.0	0.5	0.7	0.9	1.0	0.5	0.7	0.9	1.0	0.5	0.7	0.9	1.0	0.5	0.7	0.9	1.0
$\sigma_{m}^{2}/\sigma^{2}=0$																				
$\delta \backslash L^{2*}$	1.235	1.144	1.113	1.113	1.236	1.145	1.114	1.112	1.235	1.146	1.113	1.111	1.236	1.147	1.115	1.113	1.236	1.147	1.115	1.113
0.00	370.52	369.45	369.24	370.57	369.33	369.25	370.13	370.38	369.36	369.65	369.00	369.63	370.63	370.51	370.84	370.67	370.22	369.77	369.45	369.08
0.10	30.65	27.91	26.60	26.40	29.34	26.81	25.48	25.12	25.00	23.10	21.93	21.50	17.06	16.21	15.52	15.15	**5.67**	**6.08**	6.29	6.27
0.25	**8.17**	8.36	8.37	8.26	**7.80**	8.03	8.07	7.96	**6.59**	**6.93**	7.07	7.01	**4.41**	**4.89**	**5.21**	5.26	**1.47**	**2.00**	**2.39**	2.56
0.50	**2.91**	**3.44**	**3.85**	3.96	**2.78**	**3.32**	**3.72**	3.84	**2.36**	**2.90**	**3.32**	3.46	**1.60**	**2.13**	**2.53**	2.71	**1.00**	**1.00**	**1.13**	1.48
1.00	**1.12**	**1.54**	**2.02**	2.10	**1.09**	**1.47**	**1.99**	2.06	**1.03**	**1.25**	**1.87**	1.99	**1.00**	**1.01**	**1.26**	1.65	**1.00**	**1.00**	**1.00**	1.00
$\sigma_{m}^{2}/\sigma^{2}=0\mathrm{.1}$																				
$\delta \backslash L^{2*}$	1.24	1.149	1.118	1.117	1.241	1.15	1.118	1.116	1.24	1.151	1.118	1.116	1.241	1.152	1.119	1.117	1.241	1.151	1.12	1.118
0.00	370.59	370.06	370.23	369.82	369.45	370.03	369.32	369.45	369.48	369.86	370.15	370.07	370.86	370.73	369.72	369.65	370.15	370.13	370.24	369.83
0.10	30.89	28.15	26.81	26.56	29.52	27.01	25.64	25.27	25.18	23.28	22.09	21.67	17.20	16.33	15.61	15.24	**5.71**	**6.11**	6.33	6.31
0.25	**8.23**	8.42	8.43	8.31	**7.86**	8.08	8.11	8.00	**6.64**	**6.98**	7.11	7.05	**4.44**	**4.93**	**5.24**	5.28	**1.48**	**2.01**	**2.40**	2.57
0.50	**2.93**	**3.47**	**3.87**	3.98	**2.80**	**3.34**	**3.74**	3.85	**2.38**	**2.92**	**3.34**	3.47	**1.61**	**2.14**	**2.54**	2.72	**1.00**	**1.00**	**1.14**	1.49
1.00	**1.13**	**1.55**	**2.02**	2.10	**1.10**	**1.48**	**1.99**	2.06	**1.03**	**1.26**	**1.88**	2.00	**1.00**	**1.01**	**1.27**	1.67	**1.00**	**1.00**	**1.00**	1.00
$\sigma_{m}^{2}/\sigma^{2}=0\mathrm{.5}$																				
$\delta \backslash L^{2*}$	1.347	1.249	1.215	1.214	1.349	1.25	1.215	1.214	1.351	1.253	1.217	1.215	1.357	1.26	1.224	1.221	1.416	1.315	1.277	1.275
0.00	369.50	370.56	370.23	370.02	370.53	369.66	369.91	370.41	370.53	370.01	369.22	369.66	368.95	369.28	369.51	369.19	370.23	370.53	369.74	370.35
0.10	35.80	32.50	31.18	31.10	34.37	31.18	29.77	29.57	29.37	26.90	25.51	25.12	20.18	18.98	18.05	17.60	**7.18**	7.49	7.56	7.48
0.25	9.64	9.65	9.56	9.39	9.20	9.25	9.21	9.05	7.82	8.05	8.08	7.97	**5.26**	**5.71**	5.96	5.95	**1.85**	**2.39**	**2.81**	2.98
0.50	**3.43**	**3.95**	**4.33**	4.43	**3.27**	**3.80**	**4.18**	4.28	**2.79**	**3.33**	**3.73**	3.84	**1.90**	**2.43**	**2.85**	3.01	**1.00**	**1.04**	**1.53**	1.88
1.00	**1.27**	**1.77**	**2.16**	2.29	**1.22**	**1.71**	**2.11**	2.22	**1.09**	**1.48**	**1.99**	2.06	**1.00**	**1.05**	**1.57**	1.89	**1.00**	**1.00**	**1.00**	1.00
$\sigma_{m}^{2}/\sigma^{2}=1\mathrm{.0}$																				
$\delta \backslash L^{2*}$	1.594	1.477	1.437	1.436	1.599	1.482	1.441	1.439	1.618	1.5	1.458	1.456	1.681	1.559	1.515	1.512	2.135	1.979	1.925	1.924
0.00	369.92	369.53	369.29	369.31	369.98	369.95	370.25	370.05	369.09	370.37	369.66	369.89	370.56	370.35	370.68	370.28	369.21	369.91	370.18	370.77
0.10	49.08	44.30	**43.04**	43.83	47.34	42.76	**41.43**	41.83	41.57	37.44	**36.02**	36.13	30.01	27.47	26.09	25.72	14.44	13.95	13.47	13.14
0.25	13.57	13.15	12.73	12.44	12.98	12.66	12.27	11.98	11.26	11.10	10.84	10.60	**8.00**	8.23	8.24	8.12	**3.72**	**4.24**	**4.61**	4.69
0.50	**4.86**	**5.32**	**5.60**	5.64	**4.65**	**5.13**	**5.42**	5.45	**4.02**	**4.51**	**4.86**	4.93	**2.85**	**3.39**	**3.79**	3.90	**1.37**	**1.89**	**2.26**	2.43
1.00	**1.76**	**2.28**	**2.70**	2.88	**1.69**	**2.21**	**2.63**	2.80	**1.46**	**1.99**	**2.37**	2.54	**1.11**	**1.51**	**2.00**	2.08	**1.00**	**1.00**	**1.06**	1.31

Table 3 shows that as the value ρ increase, the ARL values of AIB-GWMA-ME charts decrease. However, the ARL values of AIB-GWMA-ME charts increase as $\sigma_{m}^{2}/\sigma^{\text{2}}$ increases. Specifically, for a high correlation coefficient, the detection ability of the AIB-GWMA-ME chart in detecting small shifts is better than that of the AIB-EWMA-ME chart when α ranges is 0.5 to 0.7. That is, the auxiliary variable and adjustment parameter effectively enhance the sensitivity of the GWMA framework, especially when coupled with the multiple measurements approach, further mitigating the impact of measurement error.

To investigate the effects of the constant B, Table 4 shows the ARL values of the AIB-GWMA-ME chart with multiple measurements method by the same parameters in Table 3, but with various values of B at $\sigma_{m}^{2}/\sigma^{2}=1$. Table 4 shows that as ρ≤0.75, the value of B increases, the ARL values decrease, that is, the effect of measurement error is decreasing by increasing the value of B; but it appears adverse at ρ=0.95. For example, under the case ρ=0.5 and α=0.5, the ARL decreases from 41.64 at B=1 to 29.53 at B=5, indicating that in the presence of significant measurement error, the constant B could partially compensate for the loss in detection sensitivity.

**Table 4:** ARL values for the AIB-GWMA-ME charts with multiple measurements method by taking k=5, $\sigma_{m}^{2}/\sigma^{2}=1$, q=0.95 when $ARL_{0}\approx 370$.

	ρ=0				ρ=0.25				ρ=0.5				ρ=0.75				ρ=0.95			
α	0.5	0.7	0.9	1.0	0.5	0.7	0.9	1.0	0.5	0.7	0.9	1.0	0.5	0.7	0.9	1.0	0.5	0.7	0.9	1.0
B=1																				
$\delta \backslash L^{2*}$	1.594	1.478	1.437	1.437	1.599	1.482	1.441	1.439	1.619	1.500	1.458	1.456	1.681	1.559	1.515	1.512	2.135	1.979	1.925	1.924
0.00	369.92	370.74	369.29	370.68	369.98	369.95	370.25	370.05	370.97	370.37	369.66	369.89	370.56	370.35	370.68	370.28	369.21	369.91	370.18	370.77
0.10	49.08	44.35	**43.04**	43.87	47.34	42.76	**41.43**	41.83	41.64	37.44	**36.02**	36.13	30.01	27.47	26.09	25.72	14.44	13.95	13.47	13.14
0.25	13.57	13.17	12.73	12.45	12.98	12.66	12.27	11.98	11.28	11.10	10.84	10.60	**8.00**	8.23	8.24	8.12	**3.72**	**4.24**	**4.61**	4.69
0.50	**4.86**	**5.33**	5.60	5.64	**4.65**	**5.13**	**5.42**	5.45	**4.02**	**4.51**	**4.86**	4.93	**2.85**	**3.39**	**3.79**	3.90	**1.37**	**1.89**	**2.26**	2.43
1.00	**1.76**	**2.29**	**2.70**	2.88	**1.69**	**2.21**	**2.63**	2.80	**1.46**	**1.99**	**2.37**	2.54	**1.11**	**1.51**	**2.00**	2.08	**1.00**	**1.00**	**1.06**	1.31
B=3																				
$\delta \backslash L^{2*}$	1.287	1.193	1.161	1.160	1.307	1.212	1.178	1.178	1.384	1.283	1.248	1.247	1.629	1.509	1.469	1.468	2.970	2.755	2.679	2.678
0.00	369.32	369.86	370.26	370.00	369.40	370.95	369.05	370.18	369.55	370.44	370.04	370.44	370.17	369.52	370.38	369.94	370.07	369.97	369.79	370.25
0.10	32.96	30.03	28.69	28.49	32.23	29.37	27.94	27.77	29.84	27.30	25.92	25.62	25.72	23.64	22.55	22.19	20.84	19.39	18.51	18.23
0.25	8.84	8.93	8.92	8.77	8.62	8.75	8.73	8.60	**7.94**	8.17	8.19	8.08	**6.78**	**7.09**	7.23	7.17	**5.41**	**5.84**	**6.08**	6.12
0.50	**3.15**	**3.68**	**4.06**	4.17	**3.07**	**3.61**	**3.99**	4.11	**2.83**	**3.37**	**3.77**	3.89	**2.43**	**2.95**	**3.39**	3.52	**1.96**	**2.48**	**2.91**	3.07
1.00	**1.19**	**1.65**	**2.07**	2.17	**1.16**	**1.62**	**2.05**	2.15	**1.10**	**1.50**	**2.00**	2.08	**1.03**	**1.28**	**1.90**	2.00	**1.00**	**1.07**	**1.63**	1.92
B=5																				
$\delta \backslash L^{2*}$	1.254	1.162	1.131	1.130	1.281	1.187	1.155	1.154	1.383	1.282	1.247	1.246	1.697	1.572	1.531	1.530	3.312	3.073	2.989	2.988
0.00	369.55	369.37	369.90	369.73	370.45	369.29	369.98	369.59	370.40	370.91	370.51	370.12	370.16	369.11	370.24	370.60	369.89	369.96	370.11	370.06
0.10	31.57	28.72	27.33	27.14	31.03	28.28	26.92	26.68	29.53	27.05	25.68	25.37	26.99	24.75	23.58	23.26	24.07	22.25	21.19	20.82
0.25	8.41	8.57	8.57	8.45	8.26	8.45	8.46	8.34	**7.85**	8.09	8.12	8.00	**7.13**	**7.41**	7.53	7.45	**6.30**	**6.67**	6.85	6.81
0.50	**2.99**	**3.53**	**3.93**	4.04	**2.95**	**3.48**	**3.88**	3.99	**2.80**	**3.34**	**3.75**	3.87	**2.55**	**3.08**	**3.51**	3.64	**2.27**	**2.80**	**3.23**	3.37
1.00	**1.15**	**1.58**	**2.04**	2.12	**1.13**	**1.56**	**2.02**	2.11	**1.10**	**1.48**	**1.99**	2.07	**1.05**	**1.35**	**1.93**	2.02	**1.02**	**1.20**	**1.83**	1.98

Both the AIB structure and the multiple measurements model contributed positively to monitoring effectiveness. A comparison between the GWMA-ME and AIB-GWMA-ME charts reveals that, under the same settings of B=1 and α=0.9, the ARL of GWMA-ME at ρ=0 is 43.04, which decreases to 41.43 after introducing an auxiliary variable at ρ=0.25. This result indicates that the inclusion of auxiliary information effectively shortened the average detection time. Furthermore, when the number of repeated measurements k was fixed at 5, the multiple measurements mechanism helped stabilize ARL values, preventing excessive fluctuation across different levels of measurement error.

For varying correlation coefficient ρ, the AIB-GWMA-ME chart exhibited a stable performance trend, particularly demonstrating significant improvement under high correlation case (e.g., ρ=0.95). For instance, at δ=0.10,B=1, and α=0.5, the ARL of the AIB-GWMA-ME chart is 14.44. This trend indicates that for a high correlation coefficient, the auxiliary variable effectively enhances the model’s sensitivity to small shifts and significantly mitigates the monitoring delay caused by measurement error interference.

Table 5 investigates the performance of AIB-GWMA-ME charts incorporating the multiple measurements method under various sample sizes k, correlation coefficients ρ, and adjustment parameter α. Overall, the integration of the auxiliary variables with the multiple measurements method provided positive effects on improving the stability and detection capability of the control chart, especially for large measurement error cases where it effectively suppresses the adverse impact of measurement error on detection sensitivity.

**Table 5:** ARL values for the AIB-GWMA-ME charts with multiple measurements method by taking $\sigma_{m}^{2}/\sigma^{2}=1$, q=0.95 when $ARL_{0}\approx 370$.

	ρ=0				ρ=0.25				ρ=0.5				ρ=0.75				ρ=0.95			
α	0.5	0.7	0.9	1.0	0.5	0.7	0.9	1.0	0.5	0.7	0.9	1.0	0.5	0.7	0.9	1.0	0.5	0.7	0.9	1.0
k=5																				
$\delta \backslash L^{2*}$	1.594	1.477	1.437	1.436	1.599	1.482	1.441	1.439	1.618	1.5	1.458	1.456	1.681	1.559	1.515	1.512	2.135	1.979	1.925	1.924
0.00	369.92	369.53	369.29	369.31	369.98	369.95	370.25	370.05	369.09	370.37	369.66	369.89	370.56	370.35	370.68	370.28	369.21	369.91	370.18	370.77
0.10	49.08	44.30	**43.04**	43.83	47.34	42.76	**41.43**	41.83	41.57	37.44	**36.02**	36.13	30.01	27.47	26.09	25.72	14.44	13.95	13.47	13.14
0.25	13.57	13.15	12.73	12.44	12.98	12.66	12.27	11.98	11.26	11.10	10.84	10.60	**8.00**	8.23	8.24	8.12	**3.72**	**4.24**	**4.61**	4.69
0.50	**4.86**	**5.32**	**5.60**	5.64	**4.65**	**5.13**	**5.42**	5.45	**4.02**	**4.51**	**4.86**	4.93	**2.85**	**3.39**	**3.79**	3.90	**1.37**	**1.89**	**2.26**	2.43
1.00	**1.76**	**2.28**	**2.70**	2.88	**1.69**	**2.21**	**2.63**	2.80	**1.46**	**1.99**	**2.37**	2.54	**1.11**	**1.51**	**2.00**	2.08	**1.00**	**1.00**	**1.06**	1.31
k=10																				
$\delta \backslash L^{2*}$	1.179	1.092	1.063	1.060	1.181	1.096	1.066	1.064	1.195	1.109	1.078	1.077	1.241	1.152	1.12	1.119	1.578	1.464	1.424	1.421
0.00	370.07	369.46	370.37	369.49	369.16	369.74	369.83	369.84	370.41	370.96	369.89	370.86	370.09	371.13	369.16	370.95	369.39	370.54	369.15	370.14
0.10	30.58	27.96	26.70	26.30	29.22	26.84	25.62	25.31	25.50	23.57	22.46	22.11	18.41	17.44	16.66	16.26	8.77	8.90	8.86	8.70
0.25	**8.20**	8.37	8.41	8.27	**7.84**	8.07	8.12	8.00	**6.76**	**7.10**	7.23	7.16	**4.78**	**5.24**	**5.53**	5.56	**2.25**	**2.78**	**3.21**	3.35
0.50	**2.92**	**3.45**	**3.86**	3.97	**2.79**	**3.33**	**3.74**	3.86	**2.41**	**2.95**	**3.37**	3.51	**1.72**	**2.25**	**2.67**	2.85	**1.02**	**1.19**	**1.83**	1.98
1.00	**1.12**	**1.54**	**2.02**	2.10	**1.10**	**1.48**	**1.99**	2.06	**1.03**	**1.28**	**1.89**	2.00	**1.00**	**1.02**	**1.40**	1.79	**1.00**	**1.00**	**1.00**	1.00
k=20																				
$\delta \backslash L^{2*}$	0.854	0.79	0.7685	0.767	0.856	0.793	0.771	0.769	0.865	0.801	0.780	0.779	0.898	0.831	0.811	0.809	1.144	1.058	1.029	1.027
0.00	369.88	370.77	370.78	370.64	369.60	370.37	370.22	369.04	369.65	370.70	369.66	370.73	370.93	369.06	370.50	369.19	370.15	369.81	370.73	370.12
0.10	18.93	17.76	16.97	16.55	18.17	17.07	16.32	15.91	15.72	14.91	14.38	14.02	11.21	11.00	10.82	10.57	5.28	5.68	5.94	5.94
0.25	**5.34**	**5.52**	5.71	5.66	**5.25**	**5.40**	5.55	5.47	**4.37**	**4.50**	4.61	4.51	**3.05**	**3.42**	**3.60**	3.81	**1.48**	**2.06**	**2.15**	2.30
0.50	**1.78**	**2.30**	**2.71**	2.89	**1.70**	**2.23**	**2.64**	2.81	**1.48**	**1.99**	**2.38**	2.56	**1.11**	**1.52**	**2.01**	2.08	**1.00**	**1.00**	**1.06**	1.31
1.00	**1.00**	**1.02**	**1.45**	1.82	**1.00**	**1.01**	**1.36**	1.76	**1.00**	**1.00**	**1.13**	1.47	**1.00**	**1.00**	**1.00**	1.02	**1.00**	**1.00**	**1.00**	1.00

From the perspective of the benefits of the auxiliary information and measurement error, the AIB-GWMA-ME chart significantly reduced ARL values compared to the GWMA-ME chart without auxiliary variables under the same k and parameter combinations, indicating that auxiliary information and regression-based correction mechanisms effectively enhance the detection ability to process shifts. From Table 5, under the setting k=10,ρ=0.5, and α=0.7, the ARL of the AIB-GWMA-ME chart is 7.10, which is lower than the 8.37 of the GWMA-ME chart.

Regarding the correlation coefficient ρ, the ARL values of the AIB-GWMA-ME chart decline markedly as ρ increases from 0 to 0.95, indicating a substantial improvement in detection ability when the auxiliary variable was highly correlated with the study variable. It can be observed that under k=5 and α=0.5, the ARL dropped from 49.08 (when ρ=0) to 14.44 (when ρ=0.95), demonstrating that a high correlation between the auxiliary variable and study variable leads to early detection of small shifts.

For small shift (δ=0.10), the AIB-GWMA-ME chart consistently exhibits strong detection performance across all ρ values. For instance, under k=10,ρ=0.5, and α=0.9, the ARL is 22.46; this value further decreases to 14.38 when k=20, indicating that the combination of multiple measurements and high correlation facilitated timely detection even for minor process shifts.

Table 6 presents the ARL performance of AIB-GWMA-ME charts under the linearly increasing variance model, given the settings A=0,B=1,C=0, $\sigma_{m}^{2}/\sigma^{2}=\text{1}$, and $ARL_{0}\approx 370$. The chart’s performance was evaluated across different values of variance, increased constant D, correlation coefficients ρ, and adjustment parameter α..

**Table 6:** ARL values for the AIB-GWMA-ME charts with linearly increasing variance method by taking A=0, B=1, C=0, $\sigma_{m}^{2}/\sigma^{2}=1$ when $ARL_{0}\approx 370$.

	ρ=0				ρ=0.25				ρ=0.5				ρ=0.75				ρ=0.95			
α	0.5	0.7	0.9	1.0	0.5	0.7	0.9	1.0	0.5	0.7	0.9	1.0	0.5	0.7	0.9	1.0	0.5	0.7	0.9	1.0
D=1																				
$\delta \backslash L^{3*}$	2.766	2.572	2.495	2.489	2.767	2.569	2.495	2.49	2.765	2.568	2.496	2.488	2.764	2.569	2.494	2.488	2.769	2.567	2.496	2.491
0.00	370.24	370.31	370.04	370.33	370.05	369.79	369.87	369.87	370.58	369.72	369.82	369.56	370.81	369.97	369.60	369.84	370.09	369.69	369.99	370.17
0.10	**79.84**	**75.34**	**77.00**	80.23	**76.79**	**72.83**	**73.95**	77.14	67.29	**63.68**	**64.24**	66.36	48.37	45.49	**44.76**	45.29	17.77	17.16	16.56	16.19
0.25	23.53	23.17	22.47	22.18	22.62	22.18	21.59	21.30	19.42	19.25	18.77	18.44	13.47	13.65	13.44	13.17	**4.64**	**5.22**	**5.58**	5.62
0.50	**8.75**	**9.39**	9.53	9.44	**8.39**	**9.00**	9.19	9.10	**7.18**	**7.82**	8.09	8.02	**4.92**	**5.59**	**5.95**	5.99	**1.79**	**2.29**	**2.74**	2.88
1.00	**3.31**	**3.95**	**4.41**	4.52	**3.17**	**3.80**	**4.26**	4.38	**2.74**	**3.33**	**3.81**	3.93	**1.93**	**2.45**	**2.90**	3.05	**1.04**	**1.14**	**1.45**	1.68
D=2																				
$\delta \backslash L^{3*}$	2.766	2.572	2.495	2.489	2.767	2.569	2.495	2.49	2.765	2.568	2.496	2.488	2.764	2.569	2.494	2.488	2.769	2.567	2.496	2.491
0.00	370.24	370.31	370.04	370.33	370.05	369.79	369.87	369.87	370.58	369.72	369.82	369.56	370.81	369.97	369.60	369.84	370.09	369.69	369.99	370.17
0.10	**72.50**	**71.29**	**72.72**	75.14	**70.32**	**68.88**	**70.25**	72.62	**61.67**	**60.53**	**61.33**	62.88	45.09	**43.58**	**43.34**	43.83	16.80	16.80	16.43	16.13
0.25	**21.39**	22.09	21.96	21.75	**20.58**	21.21	21.11	20.93	**17.74**	18.47	18.45	18.22	**12.50**	13.19	13.30	13.13	**4.43**	**5.15**	**5.61**	5.68
0.50	**8.09**	**9.08**	9.48	9.45	**7.78**	**8.71**	9.14	9.13	**6.72**	**7.61**	**8.06**	8.07	**4.68**	**5.50**	**5.97**	6.04	**1.85**	**2.33**	**2.79**	2.95
1.00	**3.22**	**3.92**	**4.46**	4.59	**3.11**	3.77	4.31	4.45	**2.72**	**3.33**	**3.84**	3.99	**1.98**	**2.48**	**2.95**	3.11	**1.13**	**1.25**	**1.51**	1.68
D=3																				
$\delta \backslash L^{3*}$	2.766	2.572	2.495	2.489	2.767	2.569	2.495	2.49	2.765	2.568	2.496	2.488	2.764	2.569	2.494	2.488	2.769	2.567	2.496	2.491
0.00	370.24	370.31	370.04	370.33	370.05	369.79	369.87	369.87	370.58	369.72	369.82	369.56	370.81	369.97	369.60	369.84	370.09	369.69	369.99	370.17
0.10	**65.83**	**67.59**	**69.05**	70.78	**64.14**	**65.18**	**66.79**	68.60	**56.92**	**57.54**	**58.63**	59.90	**41.90**	**41.82**	**42.04**	42.37	**15.82**	16.34	16.27	16.02
0.25	**19.59**	**21.14**	21.43	21.32	**18.83**	**20.31**	20.68	20.55	**16.36**	**17.74**	18.16	18.00	**11.65**	**12.77**	13.17	13.07	**4.24**	**5.06**	**5.63**	5.74
0.50	**7.56**	**8.83**	**9.44**	9.48	**7.28**	**8.49**	**9.10**	9.15	**6.31**	**7.43**	**8.05**	8.11	**4.48**	**5.38**	**5.98**	6.10	**1.91**	**2.35**	**2.84**	3.01
1.00	**3.15**	**3.88**	**4.48**	4.64	**3.05**	**3.74**	**4.34**	4.51	**2.69**	**3.31**	**3.87**	4.04	**2.02**	**2.50**	**2.99**	3.16	**1.21**	**1.34**	**1.57**	1.72
D=5																				
$\delta \backslash L^{3*}$	2.766	2.572	2.495	2.489	2.767	2.569	2.495	2.49	2.765	2.568	2.496	2.488	2.764	2.569	2.494	2.488	2.769	2.567	2.496	2.491
0.00	370.24	370.31	370.04	370.33	370.05	369.79	369.87	369.87	370.58	369.72	369.82	369.56	370.81	369.97	369.60	369.84	370.09	369.69	369.99	370.17
0.10	**54.82**	**60.55**	**62.73**	63.70	**53.54**	**58.63**	**60.78**	61.92	**48.10**	**52.29**	**54.07**	54.52	**36.26**	**38.66**	**39.58**	39.86	**13.89**	**15.33**	15.87	15.77
0.25	**16.63**	**19.41**	**20.53**	20.56	**15.96**	**18.68**	**19.82**	19.91	**14.09**	**16.45**	**17.50**	17.50	**10.15**	**12.01**	**12.85**	12.88	**3.89**	**4.89**	**5.64**	5.83
0.50	**6.69**	**8.32**	**9.28**	9.44	**6.46**	**8.01**	**8.98**	9.13	**5.66**	**7.08**	**7.99**	8.14	**4.12**	**5.19**	**5.98**	6.16	**1.96**	**2.40**	**2.91**	3.11
1.00	**3.02**	**3.80**	**4.52**	4.73	**2.93**	**3.67**	**4.37**	4.59	**2.63**	**3.27**	**3.92**	4.12	**2.05**	**2.52**	**3.05**	3.25	**1.30**	**1.45**	**1.68**	1.82

Table 6 shows that the ARL values of AIB-GWMA-ME charts decrease as the value of ρ increases. That is, the AIB structure continued to provide positive contributions to control chart performance. For instance, for D=1, α=0, and ρ=0, the ARL of the existing GWMA-ME chart in detecting a small shift at δ=0.10 is 77.00. Similarly, the proposed AIB-GWMA-ME chart, the ARL is reduced to 73.95 at ρ=0.25, and 16.56 at ρ=0.95, indicating that the AIB framework significantly enhances detection capability under high correlation scenarios.

Moreover, the effect of the constant D on the AIB-GWMA-ME chart reveals a generally decreasing trend in ARL values as D increases from 1 to 5 in most constant settings. For example, under ρ=0.5 and α=0.9, the ARL of AIB-GWMA-ME decreases from 64.24 at D=1 to 54.07 at D=5, indicating that linearly increasing the process variance improved the detection ability of process shifts. For the same parameter combinations, the EWMA-based structure exhibits a similar trend, though the ARL reduction was slightly more pronounced in the GWMA-based chart, suggesting that the GWMA framework was more responsive to increasing variance.

When the process experienced a small shift (δ=0.10), the AIB-GWMA-ME chart demonstrates strong detection performance, particularly when paired with appropriate parameter settings. For instance, under D=3, ρ=0.95, and α=0.5, the ARL of AIB-GWMA-ME chart is 41.82, slightly lower than 42.37 of AIB-EWMA-ME chart. It shows that the AIB-GWMA-ME chart offered marginally greater sensitivity in detecting small shifts.

A comparison between the AIB-GWMA-ME and AIB-EWMA-ME charts. Regardless of the D value, when ρ≤0.75, the AIB-GWMA-ME charts with large adjustment α=0.9 in detecting small shifts at δ=0.10 is better than the AIB-EWMA-ME charts. However, at ρ=0.95, the AIB-GWMA-ME chart performs better as D≥3 and adjustment range of 0.5 to 0.7. For instance, under D=3, ρ=0.5, and α=0.5, the ARL of AIB-GWMA-ME chart is 15.82, while that of AIB-EWMA-ME chart is 16.02.

Next, we discuss the impact of the constant C on the linearly increasing variance model. Table 7 shows the ARL values of the AIB-GWMA-ME chart with linearly increasing variance method by the same parameters in Table 6 but with different values of C keeping $\sigma_{m}^{2}/\sigma^{2}=1$,A=0,B=1,D=1.

**Table 7:** ARL values for the AIB-GWMA-ME charts with linearly increasing variance method by taking A=0, B=1, D=1, $\sigma_{m}^{2}/\sigma^{2}=1$ when $ARL_{0}\approx 370$.

	ρ=0				ρ=0.25				ρ=0.5				ρ=0.75				ρ=0.95			
α	0.5	0.7	0.9	1.0	0.5	0.7	0.9	1.0	0.5	0.7	0.9	1.0	0.5	0.7	0.9	1.0	0.5	0.7	0.9	1.0
C=0																				
$\delta \backslash L^{3*}$	2.766	2.572	2.495	2.489	2.767	2.569	2.495	2.490	2.765	2.568	2.496	2.488	2.764	2.569	2.494	2.488	2.769	2.567	2.496	2.491
0.00	370.24	370.31	370.04	370.33	370.05	369.79	369.87	369.87	370.58	369.72	369.82	369.56	370.81	369.97	369.60	369.84	370.09	369.69	369.99	370.17
0.10	**79.84**	**75.34**	**77.00**	80.23	**76.79**	**72.83**	**73.95**	77.14	67.29	**63.68**	**64.24**	66.36	48.37	45.49	**44.76**	45.29	17.77	17.16	16.56	16.19
0.25	23.53	23.17	22.47	22.18	22.62	22.18	21.59	21.30	19.42	19.25	18.77	18.44	13.47	13.65	13.44	13.17	**4.64**	**5.22**	**5.58**	5.62
0.50	**8.75**	**9.39**	9.53	9.44	**8.39**	**9.00**	9.19	9.10	**7.18**	**7.82**	8.09	8.02	**4.92**	**5.59**	**5.95**	5.99	**1.79**	**2.29**	**2.74**	2.88
1.00	**3.31**	**3.95**	**4.41**	4.52	**3.17**	**3.80**	**4.26**	4.38	**2.74**	**3.33**	**3.81**	3.93	**1.93**	**2.45**	**2.90**	3.05	**1.04**	**1.14**	**1.45**	1.68
C=1																				
$\delta \backslash L^{3*}$	2.766	2.572	2.495	2.489	2.774	2.579	2.503	2.496	2.805	2.606	2.531	2.525	2.914	2.707	2.630	2.622	3.704	3.445	3.342	3.335
0.00	370.24	370.31	370.04	370.33	369.70	369.57	369.74	369.83	370.06	369.92	369.78	369.51	370.35	370.08	369.72	369.55	370.26	369.94	369.99	370.36
0.10	**122.27**	**117.68**	**122.83**	129.37	**117.94**	**113.64**	**118.44**	124.60	**106.01**	**100.94**	**104.32**	109.50	81.32	**75.93**	**77.57**	80.68	41.81	39.24	**38.32**	38.56
0.25	39.01	36.89	**35.90**	36.05	37.46	35.50	**34.56**	34.57	32.92	31.25	30.21	29.98	24.03	23.08	22.27	21.89	11.57	11.84	11.72	11.52
0.50	14.86	14.96	14.60	14.29	14.25	14.38	14.08	13.79	12.39	12.59	12.47	12.23	**8.88**	9.31	9.41	9.27	**4.22**	**4.89**	**5.26**	5.32
1.00	**5.44**	**6.08**	**6.41**	6.42	**5.23**	**5.88**	**6.21**	6.23	**4.52**	**5.18**	**5.55**	5.60	**3.28**	**3.89**	**4.32**	4.43	**1.68**	**2.16**	**2.60**	2.75
C=2																				
$\delta \backslash L^{3*}$	2.766	2.572	2.495	2.489	2.782	2.587	2.511	2.505	2.846	2.646	2.570	2.562	3.066	2.851	2.768	2.761	4.503	4.185	4.063	4.050
0.00	370.24	370.31	370.04	370.33	369.77	370.01	370.18	370.19	369.88	369.66	370.31	369.7	369.52	370.42	370.00	369.95	370.33	370.51	370.15	369.99
0.10	**151.86**	**147.99**	**157.26**	164.40	**148.14**	**144.08**	**152.52**	160.69	**135.30**	**130.73**	**138.67**	145.3	**109.96**	**104.55**	**108.28**	114.06	70.98	**65.93**	**66.34**	68.38
0.25	51.87	48.50	**47.77**	48.42	50.13	**46.79**	**46.09**	46.83	44.72	41.69	**40.74**	41.17	34.34	32.34	31.23	31.16	20.68	20.06	19.30	18.88
0.50	20.17	19.61	18.87	18.50	19.41	18.94	18.24	17.90	17.13	16.82	16.31	15.96	12.95	13.05	12.85	12.57	**7.57**	**8.08**	8.24	8.15
1.00	**7.38**	**7.92**	8.08	8.01	**7.12**	**7.66**	7.84	7.78	6.24	6.81	7.08	7.05	**4.71**	**5.34**	**5.69**	5.72	**2.82**	**3.41**	**3.85**	3.97
C=3																				
$\delta \backslash L^{3*}$	2.766	2.572	2.495	2.489	2.788	2.593	2.517	2.511	2.879	2.677	2.598	2.590	3.180	2.956	2.869	2.861	5.033	4.678	4.539	4.528
0.00	370.24	370.31	370.04	370.33	369.72	369.80	370.19	369.77	369.75	369.62	370.09	369.66	370.26	369.60	370.28	369.72	370.20	370.14	369.91	369.80
0.10	**175.96**	**172.53**	**181.48**	189.60	**171.79**	**168.12**	**178.25**	186.02	**159.71**	**155.59**	**164.23**	172.36	**134.62**	**129.74**	**136.70**	143.81	**97.77**	**91.49**	**94.07**	98.98
0.25	63.04	**58.77**	**58.25**	59.88	61.08	**56.96**	**56.46**	57.97	55.17	**51.47**	**50.71**	51.55	44.21	41.01	**39.97**	40.40	29.68	27.96	26.83	26.59
0.50	24.83	23.74	22.76	22.42	24.01	22.98	22.10	21.76	21.51	20.74	19.93	19.53	16.89	16.59	16.05	15.68	11.10	11.31	11.17	10.95
1.00	**9.16**	9.55	9.56	9.43	**8.85**	9.26	9.30	9.17	**7.89**	**8.36**	8.46	8.36	**6.15**	**6.70**	6.95	6.93	**4.03**	**4.65**	**5.03**	5.10

It is observed from Table 7 that the values of ARL increase as the error constant C. That is, a measurement error increases with an increase in the value of C leading to suppression of detection capability. As expected, the AIB-GWMA-ME chart performs well in detecting small process shifts. Its combination of auxiliary variable and error adjustment mechanism effectively mitigated the influence of measurement error while maintaining consistent sensitivity. For example, under the setting C=1,ρ=0.75, and α=0.7, the ARL of the AIB-GWMA-ME chart is 75.93, which is lower than 80.68 of the AIB-EWMA-ME chart, demonstrating early detection of small shifts.

## 5. Simulated example

This section presents a simulated example to evaluate the detection performance of the AIB-GWMA-ME and AIB-EWMA-ME charts under the covariate model with measurement error. The constants and parameters were set as A=0, B=1, q=0.95, α=0.9 for the AIB-GWMA-ME chart and α=1.0 for the AIB-EWMA-ME chart. The control limits were defined as $L_{g}=2.495$ and $L_{e}=2.489$, respectively. A measurement error with variance $\sigma_{m}^{2}=0.1$ was incorporated, and a process mean shift of δ=0.1 was introduced after a certain point.

Table 8 displays the simulated monitoring results, including the control statistics $G_{t}$ and $E_{t}$ for AIB-GWMA-ME and AIB-EWMA-ME charts, along with their respective upper and lower control limits. As shown in the table, the AIB-GWMA-ME chart first signaled an out-of-control condition at the 44th sample, while the AIB-EWMA-ME chart detected the shift at the 46th sample. This indicates that in this particular case, the AIB-GWMA-ME chart responded slightly earlier than the AIB-EWMA-ME chart.

**Table 8:** Simulation dataset of the AIB-GWMA-ME charts with covariate method at process mean shift δ=0.1.

No.	$E_{t}$	$G_{t}$	AIB-EWMA-ME		AIB-GWMA-ME	
			LCL	UCL	LCL	UCL
1	−0.007	−0.007	–0.118	0.118	–0.103	0.103
2	0.012	0.013	–0.118	0.118	–0.103	0.103
3	−0.004	−0.005	–0.118	0.118	–0.103	0.103
4	0.000	0.000	–0.118	0.118	–0.103	0.103
5	0.024	0.024	–0.118	0.118	–0.103	0.103
6	0.022	0.019	–0.118	0.118	–0.103	0.103
7	0.033	0.030	–0.118	0.118	–0.103	0.103
8	0.055	0.050	–0.118	0.118	–0.103	0.103
9	0.069	0.060	–0.118	0.118	–0.103	0.103
10	0.050	0.039	–0.118	0.118	–0.103	0.103
11	0.057	0.047	–0.118	0.118	–0.103	0.103
12	0.057	0.046	–0.118	0.118	–0.103	0.103
13	0.074	0.063	–0.118	0.118	–0.103	0.103
14	0.061	0.048	–0.118	0.118	–0.103	0.103
15	0.048	0.036	–0.118	0.118	–0.103	0.103
16	0.054	0.044	–0.118	0.118	–0.103	0.103
17	0.057	0.046	–0.118	0.118	–0.103	0.103
18	0.048	0.038	–0.118	0.118	–0.103	0.103
19	0.049	0.039	–0.118	0.118	–0.103	0.103
20	0.063	0.054	–0.118	0.118	–0.103	0.103
21	0.068	0.058	–0.118	0.118	–0.103	0.103
22	0.050	0.039	–0.118	0.118	–0.103	0.103
23	0.037	0.029	–0.118	0.118	–0.103	0.103
24	0.048	0.041	–0.118	0.118	–0.103	0.103
25	0.047	0.039	–0.118	0.118	–0.103	0.103
26	0.059	0.052	–0.118	0.118	–0.103	0.103
27	0.034	0.026	–0.118	0.118	–0.103	0.103
28	0.068	0.063	–0.118	0.118	–0.103	0.103
29	0.089	0.080	–0.118	0.118	–0.103	0.103
30	0.082	0.071	–0.118	0.118	–0.103	0.103
31	0.071	0.059	–0.118	0.118	–0.103	0.103
32	0.082	0.071	–0.118	0.118	–0.103	0.103
33	0.070	0.058	–0.118	0.118	–0.103	0.103
34	0.069	0.059	–0.118	0.118	–0.103	0.103
35	0.078	0.068	–0.118	0.118	–0.103	0.103
36	0.088	0.077	–0.118	0.118	–0.103	0.103
37	0.092	0.081	–0.118	0.118	–0.103	0.103
38	0.104	0.091	–0.118	0.118	–0.103	0.103
39	0.090	0.076	–0.118	0.118	–0.103	0.103
40	0.089	0.076	–0.118	0.118	–0.103	0.103
41	0.087	0.075	–0.118	0.118	–0.103	0.103
42	0.088	0.076	–0.118	0.118	–0.103	0.103
43	0.106	0.095	–0.118	0.118	–0.103	0.103
44	0.117	0.104	–0.118	0.118	–0.103	0.103
45	0.117	0.101	–0.118	0.118	–0.103	0.103
46	0.123	0.108	–0.118	0.118	–0.103	0.103
47	0.147	0.130	–0.118	0.118	–0.103	0.103
48	0.135	0.115	–0.118	0.118	–0.103	0.103
49	0.116	0.098	–0.118	0.118	–0.103	0.103
50	0.125	0.108	–0.118	0.118	–0.103	0.103

**Fig 2:**
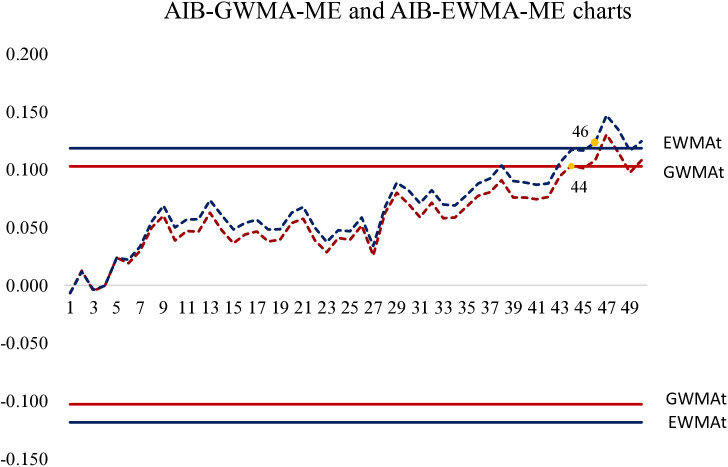
AIB-GWMA-ME and AIB-EWMA-ME charts with covariate method at process mean shift δ=0.1.

To further compare the performance of the two control charts, the results were plotted in Fig 2, which illustrates the simulated behavior of the AIB-GWMA-ME and AIB-EWMA-ME charts under the covariate model. The observation showed that the AIB-GWMA-ME chart signaled the first out-of-control condition at the 44th sample, while the AIB-EWMA-ME chart issued its signal at the 46th sample. This result indicated that both control charts demonstrated good sensitivity to small shifts. In this example, the AIB-GWMA-ME chart responded more quickly to the process mean shift, exhibiting superior detection capability and greater stability.

## 6. Conclusions

This study evaluated the detection performance of the proposed AIB-GWMA-ME chart under three types of measurement error models through Monte Carlo simulation and compared it with existing control charts, including EWMA, GWMA, AIB-EWMA, AIB-GWMA, and AIB-EWMA-ME. The AIB-GWMA-ME chart exhibited superior performance in both small and moderate shift scenarios, particularly under small shifts (δ=0.10), where its ARL values were significantly lower than those of conventional control charts. The simulation results suggest that, when applying the AIB-GWMA-ME chart, a practical approach is to use a large value of q=0.95, while choosing α in the interval 0.7≤α<1 provides a suitable alternative to improve sensitivity to small shifts and yields better performance than existing counterparts in the presence of measurement error. Notably, despite the enhanced sensitivity, the AIB-GWMA-ME chart maintained stable detection performance under in-control conditions (δ=0), without causing excessive false alarms, indicating good overall robustness.

By incorporating auxiliary variable information, the proposed control chart substantially mitigated estimation bias arising from measurement errors, while the GWMA-based weighting structure enhanced the detection ability to adapt to variation. In addition, the AIB-GWMA-ME chart exhibited a well-balanced between detection sensitivity and monitoring stability with adjustable parameters. The proposed chart provides a theoretically innovative and practically applicable tool for precision quality control in complex manufacturing environments.

### 6.1. Limitations and possible extensions of the study

When faced with the population parameters of study variable and auxiliary variable are not known in advance. One could be estimated from the reference samples. Usually, considering in-control process, generate a reference sample of 1000 observations having a bivariate normal distribution with known parameters. If all points are within the control limits, then estimate the unknown parameters further. To estimate the distribution for both the process mean and variance at each sample point, which can be time-consuming and relies on appropriate model assumptions. Moreover, the chart requires reliable auxiliary variable data since its performance may degrade if such data are unavailable or poorly correlated. This study focused solely on normally distributed data. In light of these limitations, potential directions for future research are recommended: (1) Extending the AIB-GWMA-ME chart to handle autocorrelated processes. (2) Investigating performance under non-normal or heavy-tailed data distributions. (3) Applying the proposed AIB-GWMA-ME chart to real application data to validate practical applicability.

## Supporting information

S1 Appendix Table A1. Symbols and acronyms used in this study. (DOCX)
