# Supplementary material for: Generally weighted moving average control chart in the presence of measurement error via auxiliary information utilization
Source: PLoS One. 2025 Sep 30;20(9):e0333278. doi: 10.1371/journal.pone.0333278 (PMC12483280; doi:10.1371/journal.pone.0333278)
Supplement: S1 Appendix — (DOCX) [file pone.0333278.s001.docx]

| Symbols/acronyms | Definition |
| --- | --- |
| AIB | Auxiliary Information Based |
| ARL | Average Run Length |
| EWMA | Exponentially Weighted Moving Average |
| GWMA | Generally Weighted Moving Average |
| LCL | Lower Control Limit |
| ME | Measurement Error |
| UCL | Upper Control Limit |
| $\mu$ | mean of study variable |
| $\sigma^{2}$ | variance of study variable |
| $\sigma_{m}^{2}$ | variance of random error |
| $G_{t}^{1}$ | statistic of the GWMA-ME chart |
| $\rho$ | correlation coefficient between auxiliary variable and interest variable |
| $D_{Y,t}^{1}$ | statistic of the AIB-GWMA-ME chart |
| $q$ | design parameter |
| $\alpha$ | adjustment parameter |
| $\lambda$ | smoothing constant of the EWMA chart |
